# A study to prolong breastfeeding duration: design and rationale of the Parent Infant Feeding Initiative (PIFI) randomised controlled trial

**DOI:** 10.1186/s12884-015-0601-5

**Published:** 2015-08-01

**Authors:** Bruce R. Maycock, Jane A. Scott, Yvonne L. Hauck, Sharyn K. Burns, Suzanne Robinson, Roslyn Giglia, Anita Jorgensen, Becky White, Annegrete Harries, Satvinder Dhaliwal, Peter A. Howat, Colin W. Binns

**Affiliations:** School of Public Health, Curtin University, GPO Box U1987, Perth, 6845 Australia; Collaboration for Evidence, Research and Impact in Public Health (CERIPH), Curtin University, Perth, Australia; School of Nursing and Midwifery, Curtin University, Perth, Australia; Telethon Kids Institute, Perth, Australia

## Abstract

**Background:**

Very few Australian infants are exclusively breastfed to 6 months as recommended by the World Health Organization. There is strong empirical evidence that fathers have a major impact on their partner’s decision to breastfeed and continuation of breastfeeding. Fathers want to participate in the breastfeeding decision making process and to know how they can support their partner to achieve their breastfeeding goals. The aim of the Parent Infant Feeding Initiative (PIFI) is to evaluate the effect on duration of any and exclusive breastfeeding of three breastfeeding promotion interventions of differing intensity and duration, targeted at couples but channelled through the male partner. The study will also undertake a cost-effectiveness evaluation of the interventions.

**Methods/design:**

The PIFI study is a factorial randomised controlled trial. Participants will be mothers and their male partners attending antenatal classes at selected public and private hospitals with maternity departments in Perth, Western Australia. Fathers will be randomly allocated to either the usual care control group *(CG)*, one of two medium intensity (*MI1* and *MI2*) interventions, or a high intensity (*HI*) intervention. *MI1* will include a specialised antenatal breastfeeding education session for fathers with supporting print materials. *MI2* will involve the delivery of an antenatal and postnatal social support intervention delivered via a smartphone application and *HI* will include both the specialised antenatal class and the social support intervention. Outcome data will be collected from couples at baseline and at six and 26 weeks postnatally. A total of 1600 couples will be recruited. This takes into account a 25 % attrition rate, and will detect at least a 10 % difference in the proportion of mothers breastfeeding between any two of the groups at 26 weeks at 80 % power and 5 % level of significance, using a Log-rank survival test. Multivariable survival and logistic regression analyses will be used to assess the effect of the treatment groups on the outcomes after adjusting for covariates.

**Discussion:**

The PIFI study will be the first Australian study to provide Level II evidence of the impact on breastfeeding duration of a comprehensive, multi-level, male-partner-focused breastfeeding intervention. Unique features of the intervention include its large sample size, delivery of two of the interventions by mobile device technology, a rigorous assessment of intervention fidelity and a cost-effectiveness evaluation.

**Trial registration:**

Australian New Zealand Clinical Trials Registry ACTRN12614000605695. Registered 6 June 2014

## Background

Breastfeeding is a cornerstone of both short- and long-term health [1, 2]. There are nutritional, immunological and psychological dose-related benefits for the child and as such exclusive breastfeeding can provide the greatest gains for infant development and some protection against childhood obesity and chronic disease in adult life [1–4]. Health benefits for the mother include lactational amenorrhoea, protection against ovarian and premenopausal breast cancers, bone remineralisation to levels exceeding those present before lactation and improved blood glucose profiles in women with gestational diabetes [5]. The World Health Organization and the United Nations Children’s Fund recommend exclusive breastfeeding until 6 months of age [6]. Yet nationally representative data collected in 2010 showed that while more than 95 % of Western Australian (WA) mothers initiated breastfeeding only 58 % were exclusively breastfeeding to 1 month of age and 15 % of infants were exclusively breastfed to 6 months of age [7]. Locally collected data show these rates have remained relatively unchanged for the past two decades amongst women in Perth, WA [8–10].

The decisions to breastfeed and to continue breastfeeding are influenced by a complex variety of inter-related socio-demographic, psychosocial, biomedical and environmental factors [11]. Social support for breastfeeding from a woman’s partner, family and friends has been implicated as an important factor that influences the choice and duration of breastfeeding. In Western societies in particular, there is strong evidence that fathers influence the initiation of breastfeeding [12–14], contribute to maternal breastfeeding confidence [15–18], impact decisions regarding duration and weaning [19, 20] and that without their partner’s support mothers are more likely to breastfeed for a shorter duration [21–23]. Research indicates that the support of fathers is critical to breastfeeding success and is identified as one of the strongest and most consistent factors associated with women’s willingness to breastfeed [24].

Although fathers typically describe breastfeeding as being normal and natural and can readily recite the ‘Breast is Best’ mantra, they are often poorly informed about the importance of breastfeeding and its non-equivalence with formula feeding [25]. In addition, they can hold negative attitudes regarding breastfeeding including feeling left out and fear of losing time with, and the attention of, their partner [22, 26, 27]. This highlights the need for fathers to be included in the antenatal preparation for breastfeeding [28] as well as in the postnatal period, so as to deflect negative attitudes associated with breastfeeding and increase support for the mother [29]. Furthermore, fathers want to be involved in the breastfeeding decision making process [26] and new fathers want practical advice on how they can support their partner as well as strategies for troubleshooting/problem solving common breastfeeding difficulties that their partner may encounter [30]. Fathers need to identify and fulfil their unique role in ensuring their infants receive the benefits of breastfeeding by using their knowledge and skills to assist and support their partner [29]. By involving both the mother and father in infant feeding discussions and processes, it is predicted parents will adopt more positive breastfeeding behaviours [28, 31].

While the importance of the emotional and practical support provided by a woman’s partner to her breastfeeding success is well recognised, and the involvement of fathers in breastfeeding interventions routinely called for [32], relatively few interventions have targeted fathers and have been designed to allow rigorous evaluation. A recent systematic review investigating the impact of male-partner-focused interventions on breastfeeding outcomes identified only four studies published prior to January 2012 which had used either a randomised controlled trial (RCT) or quasi experimental design [33]. Since this time, the results of a quasi-experimental study targeting couples in Viet Nam [34] and a Canadian RCT also targeting couples have been reported [35]. In addition, we have published the results of the Fathers Infant Feeding Initiative (FIFI) one of the largest (n = 700) father-focused RCT conducted to date and the first conducted in Australia [36]. FIFI and other experimental studies have been successful in increasing breastfeeding initiation rates [14, 37] and higher rates of breastfeeding in the short-term (e.g. at 6 weeks) [36, 37] amongst the intervention group but only three studies have had a significant impact on duration of exclusive breastfeeding to four [38] or six [34, 39] months. All these interventions involving fathers provided breastfeeding education classes for fathers and therefore provide sufficient empirical evidence to justify the incorporation of education sessions for fathers into the routine antenatal education program.

Our earlier intervention, the FIFI study, resulted in increased rates of any breastfeeding at six weeks but failed to result in an increase in the proportion of couples fully or exclusively breastfeeding at this time and there was no difference between intervention and control couples in the proportion still breastfeeding at six months [36]. A limitation of this earlier intervention was that it only ran for six weeks and was of relatively low intensity, involving a breastfeeding focussed antenatal education for fathers led by a male facilitator, followed by the weekly delivery of printed educational and promotional materials to six weeks postpartum.

### Aim and objectives

The aim of the PIFI study is to build on the lessons learned from the exploratory FIFI efficacy study and to design, deliver and evaluate three interventions of differing intensity and duration targeted at couples but channelled through the male partner. The main focus of the PIFI study is to prolong breastfeeding duration, and in particular the duration of exclusive breastfeeding.

#### Primary objective

To measure the effectiveness of each of three interventions designed to increase the duration of breastfeeding among new parents in urban areas.

#### Secondary objective

To determine the cost-effectiveness of each intervention.

#### Study hypotheses

It is anticipated that the intervention will result in

- *H1*: a 10 % or greater difference between the interventions and control groups in the proportion of mothers who are breastfeeding at six weeks and six months;
- *H2:* a 10 % or greater difference between the interventions and control groups in the proportion of mothers who introduce infant formula and complementary food during the first six months after birth.

## Methods/design

### Study design

The Parent Infant Feeding Initiative (PIFI) is a four arm, factorial, randomised controlled trial. The study is registered with the Australian New Zealand Clinical Trials Registry (ACTRN:12614000605695, Universal Trial No.:U1111-1155-7115). Reporting of the study will adhere to the Consolidated Standards of Reporting Trials (CONSORT) guidelines for reporting parallel group randomised trials [40].

**Fig. 1:**
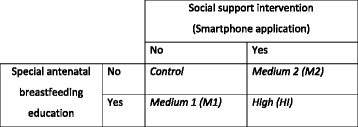
Intervention design

The trial arms will consist of a control, two medium and one high level intervention groups (see Fig. 1). The *Control Group (CG)* will have access to the usual antenatal and postnatal services provided by the hospital. The *Medium Intensity Intervention 1 (MI1)* will consist of a specialised antenatal breastfeeding education session delivered to the fathers, plus associated take-home educational print material for both parents. In the *Medium Intensity Intervention 2* (*MI2*), the social support intervention will be delivered directly to the father via a smartphone application antenatally from time of recruitment until 26 weeks postnatally. The *High Intensity Intervention* (*HI*) will include both the specialised antenatal class and the social support intervention.

### Setting

Participants will be recruited from selected public and private hospitals with maternity departments in Perth, Western Australia.

### Participants

The sample of parents will be derived from couples attending antenatal classes at one of the participating hospitals. For couples to be eligible to participate they must own a compatible smartphone, have internet access, reside within WA, both partners must intend to participate in the rearing of their child and have sufficient English language skills to engage with the intervention. Couples will be ineligible where existing medical conditions in the mother are likely to inhibit the initiation of breastfeeding or exclusive breastfeeding or if they are a same sex couple.

### Recruitment

Recruitment will take approximately 18 months, allowing for a wash-out period between each intervention arm in each hospital. The *MI1* and *HI* being hospital-based require access to antenatal classes. Usually antenatal classes run for a month and there is little overlap between classes. Each hospital has a series of antenatal classes and most metropolitan hospitals have at least 2 classes per week occurring on different days. As the researchers will be conducting the special antenatal classes and as the social support intervention happens externally to the hospital we are confident that there will be no leakage between intervention and control participants. Nevertheless, to ensure there is no contamination a washout period will be incorporated in the *MI1* and *HI* interventions.

### The interventions

The *Medium Intensity Intervention 1* (*MI1*) and the *High Intensity Intervention* (*HI*) groups will include a specialised antenatal breastfeeding education session for fathers. These sessions will be held in conjunction with antenatal classes at the participating hospitals and will require fathers to attend an additional class focusing on fatherhood, breastfeeding and anticipatory problem solving. These three key issues (importance of breastfeeding, the positive role of fathers and problem solving), were identified from the process evaluation of the FIFI study [41, 42], a review of earlier international programs and assessment of what was realistic to implement in the existing system. The class format and developed materials have been evaluated and refined during the FIFI study [42]. The educational intervention will focus on reducing fathers’ reservations about breastfeeding, reducing negative attitudes, building skills and knowledge and providing social support strategies. Specific take-home print materials to be shared by both partners will be developed that support the anticipatory problem solving component of the session. Training will be provided for the male antenatal educators in order to standardise the delivery of antenatal classes across hospitals.

From the time they are recruited, fathers randomised into either the *Medium Intensity Intervention 2* (*MI2*) or the *High Intensity Intervention* groups (*HI)* will receive sequenced, motivational, social support and educational material that will include ‘trouble shooting’ suggestions for handling common breastfeeding related difficulties (e.g. engorgement, attachment and sleep interruption), how to deal with interpersonal issues (e.g. body image and sexuality) and other life event issues (e.g. going back to work). Tips for providing practical and emotional support and caring for the mother (e.g. recognising the signs and symptoms of postnatal depression) and information on infant developmental milestones will be included. Conversational topics reinforcing these educational and motivational materials, resources and services will be ‘pushed’ out to fathers via a smartphone application from time of recruitment until 26 weeks postnatal. Push notifications delivered via the smartphone application will contain links to a library of more detailed web-based materials which fathers will be encouraged to share and discuss with their partner.

The Control Group *(CG)* will receive the standard breastfeeding support which will include information provided by hospital antenatal education programs and in-hospital breastfeeding support. All parents will be free to attend other antenatal classes or seek other breastfeeding assistance from the community. Uptake of these additional activities will be measured via the postnatal six and 26 week surveys.

### Randomisation

Randomisation will be determined through a computer-based random sequence generator. Participants will not be blinded to the true nature of the study, nor will researchers be blinded to participants’ group allocation, however the study statistician will analyse the data independently and hence will be blinded to the allocation of participants to groups.

### Ethical considerations

Participants will be provided with verbal and written information describing the purpose of the intervention and what their participation will involve. They will be advised that they may decline to participate without prejudice. Written informed consent will be obtained from both partners prior to randomisation into a study arm. They will be assigned a unique ID number which will link partners and their baseline and follow-up questionnaires.

When agreeing to participate in the study participants will be assured of anonymity and confidentiality. They will be requested to provide their name, address, email and phone number so that they can be followed-up by phone call, short message service (SMS) and email. Any woman who declines to participate in the study will be requested to anonymously provide basic demographic information, including age, years of education, marital status and intended method of feeding. It will be explained that this basic information will be used only to determine if the final sample is representative of new mothers and fathers in general. The right of non-participants to decline to provide this information will be respected.

The project has been approved by the Curtin University Human Research Ethics Committee (HR 82/2014; 14 May 2014) and the Human Research Ethics Committees responsible for the public (SCGG HREC No 2014–111; 18 Sept 2014) and private (SJGHC Ref: 777; 8 April 2015) hospital sites.

### Data collection

#### Data collection instruments

Questionnaires will be developed utilising questions previously validated in the FIFI study [36] and the first and second Perth Infant Feeding Studies [22, 43], with new items tested for face and content validity. The baseline and follow-up questionnaires will include a number of previously validated and widely used instruments to measure psychosocial factors associated with breastfeeding outcomes. These will include, but are not limited to, the Iowa Infant Feeding Attitude Scale [44], the Breastfeeding Self-Efficacy Scale [45], the Postpartum Partner Support Scale [46], the Child Care Stress Checklist [47] and the Depression Anxiety Scale (DASS-21) [48].

#### Data collection procedures

Both partners will self-complete a questionnaire at baseline and will complete follow up questionnaires either online or by telephone at six and 26 weeks postnatally. In addition, all participating mothers will complete a brief questionnaire delivered on-line or by SMS at 12 and 18 weeks to determine current breastfeeding status. Process evaluation will be conducted to assess the fidelity and quality of implementation, with an emphasis upon measuring dose and utility, and to assess the adherence and proficiency of the facilitators delivering the antenatal classes.

### Primary outcomes

- Duration of any breastfeeding
- Duration of exclusive breastfeeding

### Secondary outcomes

- Age of introduction of formula
- Age of introduction of complementary foods (‘solids’)
- Infant feeding attitudes of both partners.
- Maternal breastfeeding self-efficacy

### Statistical considerations

#### Sample size

The key outcome variable for statistical analysis is duration of breastfeeding. It is assumed that at 26 weeks, there is at least a 10 % difference in the proportion of mothers’ breastfeeding between any two of the groups. A sample size of 300 participants (fathers) is required in each of the 3 intervention groups and control group to be able to detect the difference at 80 % power and 5 % level of significance, using a Log-rank survival test. Assuming a loss to follow-up of 25 % in each group, a total of 400 participants for each group will be recruited (Total n recruited = 1600 couples, total n required =1200). The sample size was calculated in terms of the hazard ratio taking into account the censoring of data, as survival analysis will be used for the analysis of time to breastfeeding cessation, the main outcome variable.

#### Data analyses

Analyses will be performed according to the intention-to-treat (ITT) principle using data collected from all randomised participants. All participants, except those couples who experience a stillbirth or neonatal death, will be followed up regardless of their compliance with the intervention. After data are cleaned, the missing values analysis procedure in SPSS Version 22 will be used to: describe the pattern of missing data; estimate means, standard deviations, covariance and correlations for different missing value methods; and impute the missing values with estimated means from regression or expectation minimization methods.

Multivariable survival and logistic regression analyses will be used to assess the effect of the treatment groups on the outcome after adjusting for covariates. The study has a factorial treatment structure comparing Antenatal Education (Yes, No) and Social Support (Yes, No). In addition to testing the main effects, this factorial treatment structure will allow the testing of the interaction between any two factors. It is hypothesised that the combined effect of both interventions will be significantly greater than the effect of either intervention in isolation. The main effects of antenatal education and social support together with all the interactions between these factors will be assessed in the analyses of the data. Survival analysis will be used to examine the effects of the various treatment groups on the duration of any and exclusive breastfeeding. A similar analysis will be performed for introduction of infant formula and complementary food. This type of analysis allows for the presence of censored data. ‘Censored data’ refers to those cases where breastfeeding continues beyond the end of the study period or beyond the time at which the subject dropped out of the study. Variables reported in the literature to be associated with duration of overall breastfeeding and age of introduction of complementary foods will be investigated using Cox’s proportional hazards model. This model allows joint estimation of the effects of predictor variables on the ‘hazard’ – *the risk of cessation of breastfeeding/complementary food introduction*. Possible covariates that will be considered in the Survival analysis will include amongst others: socio-demographic factors such as maternal age, level of education and return to work; biomedical factors such as parity, maternal smoking and weight status; and psychosocial factors such as infant feeding attitudes, breastfeeding self-efficacy, parental stress and anxiety.

### Cost and cost effectiveness analysis

There are very few studies that have undertaken economic evaluations of breastfeeding interventions and none relating to interventions targeted at fathers [49, 50]. This study will undertake a cost-consequences analysis conducted from the perspective of the health service. The economic analysis will compare the incremental costs of the PIFI intervention (difference in costs accrued in the control arm, compared to those in the intervention arms) with those of the incremental primary outcomes, all expressed in their natural units of measurement. We will also attempt to provide cost-effectiveness data on longer term outcomes associated with breastfeeding, using existing WA epidemiological data and evidence from the literature to explore and model the linkage between breastfeeding incidence and longer term health outcomes. Sensitivity analysis will be used to explore the uncertainty in the cost and outcome data.

## Discussion

This proposal follows a *Type 2 Translational Research* approach [51] being the culmination of over 20 years of high quality observational, developmental and intervention research on breastfeeding in Australia by the research group*.* The FIFI intervention was successful in conducting foundation developmental research and implementing an exploratory efficacy study (phases 1–4 of the Phases of Research Model) as promoted by the Society for Prevention Research [52]. Given the evidence of *efficiacy* of the FIFI study [36] the next step is an *effectiveness trial* of the FIFI intervention incorporating improvements and modifications. The PIFI interventions will be informed by a relatively large body of exploratory evidence collected by us [42, 53, 54] and others [25, 30, 55] as to what fathers know and need to know about breastfeeding, and want to know with regards to supporting their partners to successfully breastfeed their infant.

The PIFI study will be the largest male-partner-focused experimental breastfeeding intervention of its kind with a predicted analysis sample of more than 1200 couples with more than 300 couples in each of the four arms of the study. The sample size is almost double that of the FIFI study [36] and much larger than earlier experimental studies which have ranged in size from less than 30 [37] to roughly 250 couples [34] in each arm of the study.

The PIFI study will employ mobile technology to deliver the intervention to participants as they go about their daily business, thus addressing an accessibility and flexibility barrier identified in the FIFI [41, 42] and other studies [25]. The PIFI study will be one of the first, if not the first, example of a breastfeeding ecological momentary intervention (EMI) targeting fathers. The term EMI is described by Heron and Smyth as “providing a framework for treatments characterised by the delivery of interventions to people as they go about their daily lives….. these interventions are *ecologically* valid because they occur in the natural environment, and are provided at specifically identified *moments* in everyday life” [56, p2].

The use of digital platforms such as the internet and mobile devices (e.g. smartphones, palmtop computers and tablets) to deliver health behaviour interventions is increasing rapidly [56–58]. Smartphones in particular, offer specific benefits in terms of engagement with users [59]. A review of the breastfeeding literature found however, only one quasi-experimental intervention in China which assessed the impact of an SMS intervention on infant feeding practices [60]. Similarly, a recent systematic review by our group [61] identified only one quasi-experimental intervention which had employed Internet delivery to provide breastfeeding information and support, and reported on breastfeeding outcomes.

Many studies reporting intervention effectiveness fail to consider or discuss intervention fidelity [62], which is the extent to which the intervention was delivered in the manner it was intended [63]. The reporting of intervention fidelity is now included in the CONSORT guidelines for reporting of randomised trials of non-pharmacologic treatment [64] and as such the principles of fidelity measurement will be embedded within the design stage of the PIFI interventions and the training and assessment of those delivering the specialised antenatal classes for fathers [65]. Strategies for ensuring intervention fidelity will be implemented and assessed and will address the five basic components of intervention fidelity as identified by Murphy and Gutman [62, p387]. These being 1) intervention design; 2) training of providers; 3) intervention delivery; 4) receipt of intervention; and 5) enactment of skills gained from the intervention.

This project will also undertake both a cost and cost-effectiveness analysis of the interventions under study. Whilst studies have demonstrated the increased costs of formula feeding in relation to cost of excess illness [66, 67], there is limited evidence on the cost and cost-effectiveness of interventions aimed at increasing the uptake and duration of breastfeeding [49, 68]. The limited research currently available suggests that while high level interventions may have more significant results, when costs are considered moderate interventions can be more sustainable and can also produce significant changes. This research will examine the cost and benefits associated with the different levels of intervention and make suggestions with regard to which of these is more cost-effective. There is agreement in the literature that further research on the cost and cost-effectiveness is important in this area [49, 50, 69].

To our knowledge, the PIFI study will be the first Australian study to provide Level II evidence of the impact on breastfeeding duration of a comprehensive, multi-level, male-partner-focused breastfeeding intervention. Potential beneficial outcomes include increased duration of breastfeeding, delayed use of infant formula and complementary foods, a better start to life for the infants and less chronic disease later in life.

## Abbreviations

ANZCTR: Australian New Zealand Clinical Trials Registry
EMI: Ecological momentary intervention
FIFI: Fathers infant feeding initiative
PIFI: Parent infant feeding initiative
RCT: Randomised controlled trial
SMS: Short message service
SPSS: Statistical package for the social sciences
UTN: Universal trial number
WA: Western Australia
